# A multimodal framework for attenuation of piston and planar waves in impedance-lined ducts

**DOI:** 10.1371/journal.pone.0339029

**Published:** 2025-12-29

**Authors:** Abdulwahed Alrashdi, Muhammad Safdar, Hafiza Umara Ismail, Naif Alkuhayli

**Affiliations:** 1 Department of Mathematics, College of Science, Jouf University, Sakaka, Saudi Arabia; 2 Department of Mathematics, Quaid-i-Azam University, Islamabad, Pakistan; 3 Department of Mathematics, Capital University of Science and Technology, Islamabad, Pakistan; COMSATS University Islamabad, PAKISTAN

## Abstract

This study presents a multimodal formulation to investigate the scattering of acoustic waves in duct systems lined with locally reacting liners. The primary objective is to analyze the attenuation of piston-driven and planar acoustic radiations by employing impedance-based boundary conditions that accurately model liner behavior. A multimodal framework is developed to solve the governing boundary value problems by projecting acoustic fields onto orthogonal basis functions, with eigenvalues and eigenvectors used to characterize the modal propagation. The proposed method is validated against benchmark configurations, including rigid-walled ducts and ducts with impedance boundaries, and cross-compared with traditional mode-matching techniques. Numerical results demonstrate the effectiveness of the liners in attenuating acoustic energy, particularly at low frequencies, and confirm the convergence of the multimodal approach across a range of excitation conditions. The formulation is further applied to a reactive silencer geometry containing impedance-lined cavities, highlighting the liner’s influence on wave scattering and overall noise reduction performance. This work provides a comprehensive modeling framework for evaluating liner treatments in complex acoustic systems and contributes to the design of efficient noise-control devices in ducts and silencers.

## 1 Introduction

Acoustic liners [1] are widely employed to mitigate noise generated by Heating, Ventilation, and Air Conditioning (HVAC) systems in buildings, as well as in chimney stacks, power plants, vehicles, and aircraft. Two widely used classes are *locally reacting* and *bulk reacting* liners. Locally reacting liners typically comprise perforated facesheets backed by honeycomb cavities and primarily support wave motion normal to the duct walls; bulk reacting liners are porous media that provide distributed dissipation across the layer. Locally reacting designs are effective over a tunable low–to–mid-frequency band and are valued for robustness and high acoustic resistance, whereas bulk treatments often offer broader-band absorption but are comparatively less efficient at very low frequencies. Achieving efficient noise control at low frequencies remains a significant challenge in acoustic design. In this work, we focus on locally reacting liners because their frequency-dependent surface-impedance model couples directly to our multimodal/mode-matching formulation and reflects configurations commonly used in practical lined-duct silencers; bulk-reacting cases, while important for broadband control, lie beyond our present scope.

Propagation of sound in lined ducts with nominally rigid walls has been extensively investigated (e.g., [2,3]). Analytical and numerical frameworks that accommodate impedance boundary conditions include mode-matching [4–7], the finite element method (FEM) [8], and point-matching [9], each with trade-offs set by geometry, frequency, and modeling objectives. Félix and Pagneux [10] treated an adiabatic, lossy medium in a curved circular duct with reacting boundaries and, via a multimodal expansion, computed velocity and pressure fields subject to impedance conditions. Similarly, Bi et al. [11] investigated a circular rigid duct lined with non-uniform, piecewise-constant reacting liners. Using a multimodal technique, they linked duct modes to the eigenpairs of a transformed system and demonstrated applicability across low, mid, and high frequencies, with only minor performance differences due to liner non-uniformity. Kirby et al. [12] studied exhaust-system silencers that combine reactive and dissipative elements and showed improved high-frequency performance when both are incorporated. The Mode-matching technique has been extensively employed by researchers to analyze energy scattering in both active and passive liner systems (see [13–19]).

This study investigates the reflection and transmission of acoustic waves in a channel lined with locally reacting liners. A multimodal scheme is employed to analyze the scattering behavior of piston-driven and planar incident waves in the presence of such liners. The propagation characteristics of the modes are determined through the eigenvalues and eigenvectors of the transformed system. The primary aim of this study is to develop and validate a multimodal formulation for modeling wave scattering in duct systems lined with acoustic treatments. Specifically, the objective is to evaluate the impact of locally reacting liners on the attenuation of piston and planar acoustic radiations. The study also seeks to benchmark the proposed approach against established mode-matching techniques in canonical duct configurations, and to extend its applicability to complex silencer structures with impedance-lined cavities. Relevant prior work includes the study by Sirotto et al. [20], which examined far-field noise mitigation from a subsonic jet interacting with acoustic liners. Their integrated experimental and numerical approach utilized a wavepacket-based jet noise source and impedance boundary conditions. Recently, Alruwaili et al. [21] used mode-matching techniques with generalized eigenfunction properties to address structure-borne radiation attenuation in a chamber with lined walls. Similarly, Alkuhayli et al. [22] analyzed the effect of liners on planar radiations from bifurcated inlet-outlet systems.

The present work emphasizes the attenuation of both piston and planar radiations using liner treatments. The multimodal formulation is adopted to solve the associated boundary value problems by projecting the solution onto orthogonal basis functions. Impedance-based projection techniques, integrated with the multimodal framework, are detailed in [23–28]. To validate the extracted modal solutions, two benchmark duct configurations are considered: (i) a rigid-walled duct excited by a plane piston along one boundary, and (ii) a similar configuration where the upper rigid wall is replaced by an impedance boundary. Both are analyzed using the multimodal and mode-matching methods. The accuracy of the multimodal approach is assessed by comparing modal coefficients for varying piston velocities. Subsequently, the methodology is extended to model the influence of locally reacting liners within cavity structures. By treating the liners as integral components of a reactive silencer, the study explores acoustic wave scattering in a silencer chamber lined with impedance materials. The structure of the article is as follows: Sect 2 presents the modal analysis of piston-driven acoustic fields in canonical ducts. Sect 3 describes the application of the multimodal formulation to evaluate the influence of liner conditions in a waveguide. Sect 4 provides numerical results along with a discussion, while Sect 5 concludes the article with a summary and final remarks.

## 2 Modal techniques for piston-driven acoustic fields in canonical ducts

This section contains the Multi-modal and Mode-matching formulations by considering two archetypal waveguide problems. We first analyse piston-generated sound in a duct whose horizontal and vertical walls are entirely rigid (rigid-rigid case); we then repeat the study for an otherwise rigid duct whose upper boundary incorporates an impedance treatment (rigid-impedance case). In each configuration the governing boundary-value problem is solved with the Multi-modal method to obtain the propagating acoustic modes, and the results are cross-checked against those produced by the analytical Mode-Matching approach based on variable separation.

**Fig 1:**
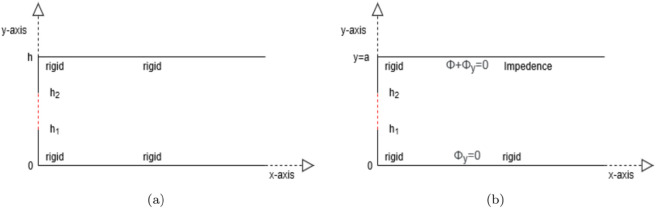
Canonical duct geometries used to benchmark the modal techniques. The red dashed line section expresses the position piston, where, (a) rigid-rigid duct and (b) rigid-impedance duct.

The physical domain is a two-dimensional rectangular duct lying in the *xy*-plane between *y* = 0 and *y* = *h* (Fig 1). In the first setup (Fig 1(a)) both horizontal walls at *y* = 0 and *y* = *h* are acoustically rigid, and the left boundary at *x* = 0 is rigid except for the segment $h_{1}<y<h_{2}$ (highlighted by red dashed lines), which acts as an open or reactive window through which acoustic energy may enter or leave the duct. The second setup (Fig 1(b)) represents a more realistic mixed-boundary system often encountered in mufflers, HVAC ducts, or other noise-control devices. Here the lower wall at *y* = 0 and large portions of the vertical and upper walls remain rigid, whereas the same segment $h_{1}<y<h_{2}$ on the left wall is treated with an impedance boundary. This mixed condition modifies the scattering and resonance characteristics of the guide. In both cases the duct is filled with a compressible fluid of uniform density ρ and sound speed *c*.

The propagation of acoustic waves within the duct is governed by the classical wave equation for the acoustic velocity potential $\bar{\Phi }(\bar{x},\bar{y},\bar{t})$:

$$\frac{\partial^{2}\bar{\Phi }}{\partial {\bar{x}}^{2}}+\frac{\partial^{2}\bar{\Phi }}{\partial {\bar{y}}^{2}}=\frac{1}{c^{2}}\frac{\partial^{2}\bar{\Phi }}{\partial {\bar{t}}^{2}}.$$ (1)

The acoustic pressure $\bar{p}$ and particle velocity $\bar{v}$ are related to the potential by $\bar{p}=-\rho \;\partial \bar{\Phi }/\partial \bar{t}$ and $\bar{v}=\nabla \bar{\Phi }$, respectively. Assuming harmonic time dependence of the form $e^{-i\omega \bar{t}}$, the potential is decomposed as:

$$\bar{\Phi }(\bar{x},\bar{y},\bar{t})=\bar{\phi }(\bar{x},\bar{y})\;e^{-i\omega \bar{t}},$$ (2)

where $\bar{\phi }(\bar{x},\bar{y})$ represents the time-independent spatial part. Substituting (2) into (1) yields the two-dimensional Helmholtz equation

$$\frac{\partial^{2}\bar{\phi }}{\partial {\bar{x}}^{2}}+\frac{\partial^{2}\bar{\phi }}{\partial {\bar{y}}^{2}}+k^{2}\bar{\phi }=0,$$ (3)

where k=ω/c is the wavenumber. For convenience, we nondimensionalize the problem using the characteristic length *k*^−1^ and time scale $\omega^{-1}$ such that


$$r=k\bar{r},\;z=k\bar{z},\;\phi =\frac{k^{2}}{\omega }\bar{\phi }.$$

In these variables, the Helmholtz equation reduces to [28]

$$(\nabla^{2}+1)\;\phi =0.$$ (4)

The boundary conditions and modal solutions for both the ducts are explained subsequently.

### 2.1 Rigid-rigid configuration.

We examine a semi-infinite duct that is excited by a plane piston located at *x* = 0, as illustrated in Fig 1. All boundaries of the duct are considered acoustically rigid. Under this assumption, the normal component of the acoustic velocity vanishes on the rigid walls, leading to the following boundary conditions:

$$\frac{\partial \phi }{\partial y}=0,\;y=\{0,h\},\;0<x<\infty ,$$ (5)

$$\frac{\partial \phi }{\partial x}=0,\;x=0,\;0<y<h_{1}\;\text{and}\;h_{2}<y<h.$$ (6)

The acoustic excitation is applied by a piston placed along the wall section $h_{1}\leq y\leq h_{2}$ at *x* = 0, oscillating with a uniform velocity amplitude *U*. This gives rise to the boundary condition at the source interface:

$$\frac{\partial \phi }{\partial x}=\left\{ \begin{array}{cc} 0, & 0\leq y<h_{1},\\ U, & h_{1}\leq y\leq h_{2},\\ 0, & h_{2}<y\leq h. \end{array}\right.$$ (7)

To analyze wave propagation within the duct, both the *mode-matching* and *multi-modal* methods are employed, with details provided in the following sections.

#### 2.1.1 Mode-matching approach.

To derive the modal solution, we apply the method of separation of variables to the dimensionless Helmholtz equation (4) subject to the boundary conditions (5)–(6) by assuming ϕ(x,y)=X(x)Y(y). This yields a set of ordinary differential equations whose solutions in the transverse direction are:


$$Y_{n}(y)=\cos(\tau_{n}y),\;n=0,1,2,\ldots ,$$

where $X_{n}(x)=e^{i\eta_{n}x}$, in which $\eta_{n}=\sqrt{1-\tau_{n}^{2}}$ express the axial wavenumbers such that $\tau_{n}=\frac{n\pi }{h}$. The eigen functions satisfy orthogonality over the duct height and are normalized according to:

$$\int_{0}^{h}\psi_{m}(y)\psi_{n}(y)\;dy=\delta_{mn},$$ (8)

with the normalized basis functions defined as:


$$\psi_{n}(y)=\Lambda_{n}\cos(\tau_{n}y),\;\text{where}\;\Lambda_{n}=\left\{ \begin{array}{cc} \frac{1}{\sqrt{h}}, & n=0,\\ \sqrt{\frac{2}{h}}, & n\geq 1. \end{array}\right.$$

The general solution for waves propagating in the positive *x*-direction is then expressed as:

$$\phi (x,y)=\sum_{n=0}^{\infty }A_{n}\psi_{n}(y)e^{i\eta_{n}x},$$ (9)

where *A*_*n*_ denotes the modal amplitude to be determined from the boundary conditions. To evaluate *A*_*n*_, we impose the excitation condition at *x* = 0, as given in Eq (7). Multiplying both sides by $\psi_{m}(y)$ and integrating over y∈[0,h], we substitute Eq (9) and apply the orthonormality condition (8), leading to:

$$i\sum_{n=0}^{\infty }A_{n}\eta_{n}\int_{0}^{h}\psi_{n}(y)\psi_{m}(y)\;dy=U\int_{h_{1}}^{h_{2}}\psi_{m}(y)\;dy.$$ (10)

This results in the closed-form expression for the modal amplitudes:

$$A_{m}=\frac{-iU}{\eta_{m}}Q_{m},$$ (11)

where the projection coefficient *Q*_*m*_ is defined as:


$$Q_{m}=\int_{h_{1}}^{h_{2}}\psi_{m}(y)\;dy=\left\{ \begin{array}{cc} \frac{h_{2}-h_{1}}{\sqrt{h}}, & m=0,\\ \sqrt{\frac{2}{h}}\cdot \frac{2}{m\pi }\left[\sin\left(\frac{h_{2}\pi }{h}\right)-\sin\left(\frac{h_{1}\pi }{h}\right)\right], & m\geq 1, \end{array}\right.$$

and it defines the coupling of modal amplitudes *A*_*n*_ with the piston radiations. In the next section, a multi-modal approach will be applied to further investigate wave behavior under more complex boundary configurations.

#### 2.1.2 Multimodal approach.

In this section, we derive the traveling wave solution using the multimodal expansion technique. To this end, we assume the following form for the potential field:

$$\phi (x,y)=\sum_{n=0}^{\infty }A_{n}Z_{n}(y)e^{is_{n}x},$$ (12)

where the coefficients *A*_*n*_, mode shapes *Z*_*n*_(*y*), and axial wavenumbers *s*_*n*_ are unknown and need to be determined. Substituting the expansion (12) into the Helmholtz equation (4) and applying the rigid-wall boundary conditions (5), we obtain the following ordinary differential equation:

$$\frac{d^{2}Z_{n}}{dy^{2}}+\gamma_{n}^{2}Z_{n}=0,$$ (13)

subject to Neumann boundary conditions:

$$Z_{n}^{\prime }(y)=0,\;y\in \{0,h\},$$ (14)

where $\gamma_{n}^{2}=1\;-\;s_{n}^{2}$ denotes the square of the transverse wavenumber. To proceed, we consider the associated eigenvalue problem:

$$\frac{d^{2}\xi_{n}(y)}{dy^{2}}+\alpha_{n}^{2}\xi_{n}(y)=0,$$ (15)

with the same boundary conditions:

$$\xi_{n}^{\prime }(y)=0,\;y\in \{0,h\}.$$ (16)

The eigenfunctions $\xi_{n}(y)$ form an orthonormal basis over the interval [0,*h*], satisfying the orthogonality condition:

$$\int_{0}^{h}\xi_{n}(y)\xi_{m}(y)\;dy=\delta_{mn}.$$ (17)

The corresponding eigenvalues are $\alpha_{n}=\frac{n\pi }{h}$, and the normalized eigenfunctions are:


$$\xi_{n}(y)=\Lambda_{n}\cos(\alpha_{n}y),\;\text{with}\;\Lambda_{n}=\left\{ \begin{array}{cc} \frac{1}{\sqrt{h}}, & n=0,\\ \sqrt{\frac{2}{h}}, & n\geq 1. \end{array}\right.$$

We now obtain the solution by projecting onto the orthogonal basis $\{\xi_{m}(y){\}}_{m=0}^{\infty }$. Since these basis functions satisfy the rigid-wall boundary conditions, we expand *Z*_*n*_(*y*) as:

$$Z_{n}(y)=\sum_{m=0}^{\infty }B_{nm}\xi_{m}(y)=\xi_{n}^{\;t}B_{n}^{\;t},$$ (18)

where


$$\xi_{n}^{\;t}=[\begin{array}{ccccc} \xi_{n0} & \xi_{n1} & \ldots & \xi_{nm} & \ldots \end{array}],\;B_{n}^{\;t}=[\begin{array}{ccccc} B_{n0} & B_{n1} & \ldots & B_{nm} & \ldots \end{array}].$$

Multiplying Eq (13) by $\xi_{n}$ and integrating over the domain y∈[0,h], we obtain:

$$\int_{0}^{h}\xi_{n}\frac{d^{2}Z_{n}}{dy^{2}}\;dy+\gamma_{n}^{2}\int_{0}^{h}\xi_{n}Z_{n}\;dy=0.$$ (19)

Applying integration by parts to the first term and using the boundary conditions (14) and (16), we get:

$$\int_{0}^{h}\xi_{n}\frac{d^{2}Z_{n}}{dy^{2}}\;dy=\int_{0}^{h}Z_{n}\frac{d^{2}\xi_{n}}{dy^{2}}\;dy.$$ (20)

Substituting into (19) yields:

$$\int_{0}^{h}Z_{n}\frac{d^{2}\xi_{n}}{dy^{2}}\;dy+\gamma_{n}^{2}\int_{0}^{h}\xi_{n}Z_{n}\;dy=0.$$ (21)

Inserting the expansion (18) and using the orthogonality of the eigenfunctions, we derive the following algebraic system:

$$\sum_{p=0}^{\infty }\left({\left(\frac{p\pi }{h}\right)}^{2}+\gamma_{n}^{2}\right)B_{np}\delta_{pm}=0,\;m=0,1,2,\ldots$$ (22)

This can be written in matrix form as:

$$N_{1}B+\gamma_{n}^{2}IB=0,$$ (23)

where *N*_1_ is a diagonal matrix of squared eigenvalues:


$$N_{1}=(\begin{array}{cccc} 0 & 0 & 0 & \ldots \\ 0 & \frac{\pi^{2}}{h^{2}} & 0 & \ldots \\ 0 & 0 & \frac{4\pi^{2}}{h^{2}} & \ldots \\ \vdots & \vdots & \vdots & \ddots \end{array}).$$

The eigenvalues $\gamma_{n}^{2}$ characterize the transverse modal behavior. The full operator matrix becomes $N=k^{2}I\;+\;N_{1}$, and the corresponding eigenvectors are denoted by *X*. Consequently, the field potential can be written in compact modal form as

$$\phi (x,y)=\xi^{t}XD(x)A,$$ (24)

where $\xi^{t}$ is the row vector of basis functions, A is the vector of modal amplitudes, and *D*(*x*) is the diagonal matrix of traveling wave factors:


$$D(x)=(\begin{array}{cccc} e^{is_{0}x} & 0 & 0 & \ldots \\ 0 & e^{is_{1}x} & 0 & \ldots \\ 0 & 0 & e^{is_{2}x} & \ldots \\ \vdots & \vdots & \vdots & \ddots \end{array}),\;A^{t}=[\begin{array}{cccc} A_{0} & A_{1} & A_{2} & \ldots \end{array}].$$

To determine A, we differentiate (24) with respect to *x* and match it with the velocity boundary condition (7):

$$\xi^{t}X{\frac{d}{dx}D(x)|}_{x=0}A=\left\{ \begin{array}{cc} 0, & 0\leq y<h_{1},\\ U, & h_{1}\leq y\leq h_{2},\\ 0, & h_{2}<y\leq h. \end{array}\right.$$ (25)

Multiplying both sides by ξ and integrating over [0,*h*], we obtain:

$$\int_{0}^{h}\xi \xi^{t}dy\cdot X{\frac{d}{dx}D(x)|}_{x=0}A=U\int_{h_{1}}^{h_{2}}\xi (y)dy.$$ (26)

Solving this system leads to the explicit form of the amplitude vector:

$$A=iD_{0}^{-1}X^{-1}QU,\;\text{with}\;Q=\int_{h_{1}}^{h_{2}}\xi (y)dy,$$ (27)

where $D_{0}={\frac{d}{dx}D(x)|}_{x=0}$ is a diagonal matrix with entries *is*_*n*_.

### 2.2 Rigid-impedance configuration

In this configuration, the lower wall at *y* = 0 is acoustically rigid, while the upper wall at *y* = *h* satisfies an impedance boundary condition. The corresponding boundary conditions for the semi-infinite duct are expressed as:

$$\frac{\partial \phi }{\partial y}=0,\;y=0,\;0<x<\infty ,$$ (28)

$$\phi +\frac{\partial \phi }{\partial y}=0,\;y=h,\;0<x<\infty .$$ (29)

The schematic representation of the duct geometry, featuring rigid and impedance surfaces, is shown in Fig 1(b). The acoustic wave propagation in this setup can be analyzed using both the mode-matching and multimodal methods, which are elaborated below.

#### 2.2.1 Mode-matching solution.

Following the separation of variables approach outlined in Sect 2.2.1, the wave modes are represented by the eigenfunctions


$$Y_{n}(y)=\cos(\tau_{n}y),\;n=0,1,2,\ldots ,$$

which satisfy the orthogonality condition

$$\int_{0}^{h}\psi_{m}(y)\psi_{n}(y)\;dy=\delta_{mn},$$ (30)

with the normalized form


$$\psi_{m}(y)=\sqrt{\frac{2}{h}}\cos(\tau_{m}y),\;\tau_{m}=\frac{m\pi }{h}.$$

To determine the modal amplitudes *A*_*m*_, we substitute Eq (9) into boundary condition (7), multiply by $\psi_{m}(y)$, and integrate over y∈[0,h], resulting in:

$$A_{m}=\frac{-iU}{\eta_{m}}Q_{m},$$ (31)

where


$$Q_{m}=\int_{h_{1}}^{h_{2}}\psi_{m}(y)\;dy=\sqrt{\frac{2}{h}}\left\{\sin(\tau_{m}h_{2})-\sin(\tau_{m}h_{1})\right\}.$$

#### 2.2.2 Multimodal approach.

For the multimodal formulation, Eq (12) is substituted into the Helmholtz equation (4), and the boundary conditions (28)–(29) are enforced, yielding:

$$\frac{d^{2}Z_{n}}{dy^{2}}+\gamma_{n}^{2}Z_{n}=0,$$ (32)

with boundary conditions:

$$Z_{n}^{\prime }(0)=0,\;Z_{n}(h)+\alpha Z_{n}^{\prime }(h)=0,$$ (33)

where α is an arbitrary parameter that can used to link the characteristic impedance of the liner surface. Multiplying Eq (32) by the orthogonal function $\xi_{n}$ and integrating over [0,*h*], we obtain:

$$\int_{0}^{h}\xi_{n}\frac{d^{2}Z_{n}}{dy^{2}}\;dy+\gamma_{n}^{2}\int_{0}^{h}\xi_{n}Z_{n}\;dy=0.$$ (34)

Using integration by parts and applying boundary conditions (16) and (33), the first integral evaluates to:

$$\int_{0}^{h}\xi_{n}\frac{d^{2}Z_{n}}{dy^{2}}\;dy=-\frac{1}{\alpha }\xi_{n}(h)Z_{n}(h)+\int_{0}^{h}Z_{n}\frac{d^{2}\xi_{n}}{dy^{2}}\;dy.$$ (35)

Substituting (35) into (34) yields:

$$-\frac{1}{\alpha }\xi_{n}(h)Z_{n}(h)+\int_{0}^{h}Z_{n}\frac{d^{2}\xi_{n}}{dy^{2}}\;dy+\gamma_{n}^{2}\int_{0}^{h}\xi_{n}Z_{n}\;dy=0.$$ (36)

Using the expansion $\xi_{n}=\xi_{nm}=\Lambda_{m}\cos\left(\frac{m\pi y}{h}\right)$, and Eq (18), the relation becomes:

$$-\frac{1}{\alpha }\sum_{p=0}^{\infty }B_{np}(h)\xi_{np}(h)\xi_{nm}(h)-\sum_{p=0}^{\infty }{\left(\frac{m\pi }{h}\right)}^{2}B_{np}\delta_{pm}+\gamma_{n}^{2}\sum_{p=0}^{\infty }B_{np}\delta_{pm}=0.$$ (37)

This leads to the matrix representation:

$$-\frac{1}{\alpha }N_{1}B-N_{2}IB+\gamma_{n}^{2}IB=0,$$ (38)

with


$$N_{1}=(\begin{array}{ccc} \xi_{0n}(h)\xi_{0n}(h) & \xi_{0n}(h)\xi_{1n}(h) & \cdots \\ \xi_{1n}(h)\xi_{0n}(h) & \xi_{1n}(h)\xi_{1n}(h) & \cdots \\ \vdots & \vdots & \ddots \end{array}),\;N_{2}=(\begin{array}{ccc} 0 & 0 & \cdots \\ 0 & \frac{\pi^{2}}{h^{2}} & \cdots \\ \vdots & \vdots & \ddots \end{array}).$$

Define the operator


$$N=Ik^{2}-\frac{1}{\alpha }N_{1}-N_{2}I,$$

so that its eigenvalues yield $\gamma_{n}^{2}$, and the associated eigenvectors define the modal matrix *X*. The potential field is then expressed as:

$$\phi (x,y)=\xi^{t}XD(x)A,$$ (39)

where *D*(*x*) is a diagonal matrix with entries $e^{is_{n}x}$. Finally, the modal amplitude vector A is given by:

$$A=iD_{0}^{-1}X^{-1}QU,$$ (40)

where *D*_0_ is the diagonal matrix of propagation constants *is*_*n*_.

## 3 Multimodal analysis of waveguides with reactive liner cavities

**Fig 2:**
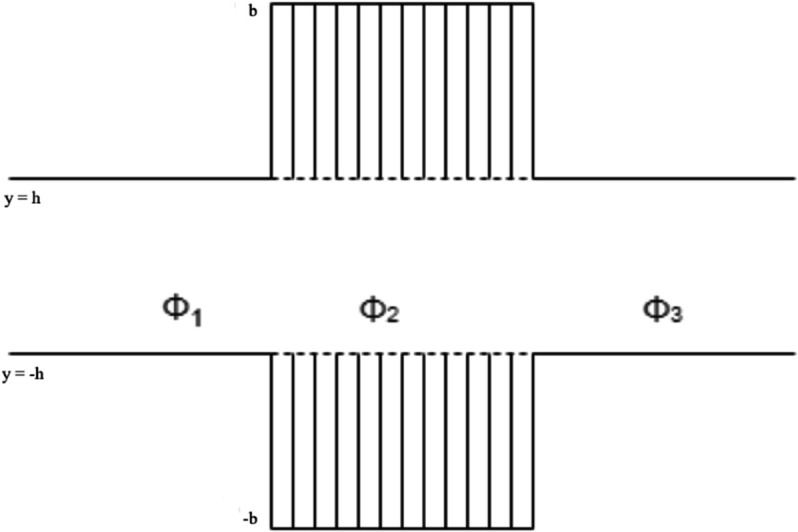
The geometrical configuration of the waveguide.

In this section, we investigate a waveguide system incorporating reactive linings within its central region. The setup involves wave propagation in a duct bounded by both rigid and impedance surfaces. Specifically, we analyze the acoustic response in a double-cavity configuration composed of honeycomb layers. The impedance boundary conditions are formulated using the field potential and the continuity of velocity. The governing boundary value problem is solved using the multi-modal method. Propagation and scattering characteristics of traveling waves in the inlet and outlet regions are examined. The reflection and transmission coefficients are obtained by enforcing continuity of pressure and velocity at the interfaces. Numerical results demonstrating wave behavior are presented at the end of this section. Consider a plane acoustic wave propagating from the negative *x*-direction toward *x* = 0 in an infinite waveguide. Fig 2 illustrates a two-dimensional waveguide geometry, confined between two horizontal lines at *y* = *h* and *y* = −*h*. At the interface *x* = 0, the incident wave gives rise to a spectrum of reflected and transmitted modes propagating in various directions. The central portion of the waveguide contains an embedded segment of finite width 2*b*, symmetrically placed about the vertical axis and partially spanning the vertical extent between the upper and lower boundaries. The waveguide is divided into three regions: Region I (*x*<–*b*), where the acoustic field $\Phi_{1}$ consists of incident and reflected waves; Region II (–*b*<*x*<*b*), the central region lined with locally reacting acoustic material, represented by $\Phi_{2}$, where wave attenuation occurs; and Region III (*x*>*b*), where the transmitted wave field $\Phi_{3}$ exists. The entire waveguide is filled with a compressible fluid of density ρ and sound speed *c*, allowing acoustic wave propagation throughout.

For clarity, the total field potential Φ(x,y) is defined piecewise across the three regions as:

$$\Phi (x,y)=\left\{ \begin{array}{cc} \phi_{1}(x,y), & \;-\infty <x<-L,\;-h<y<h,\\ \phi_{2}(x,y), & \;-L<x<L,\;-h<y<h,\\ \phi_{3}(x,y), & \;L<x<\infty ,\;-h<y<h \end{array}\right..$$ (41)

### 3.1 Impedance conditions for reactive liners

To derive the conditions associated with the reactive liners embedded in the second region of the waveguide, the acoustic impedance is defined as

$$ℨ(y)=\frac{p}{𝐯\cdot 𝐧},$$ (42)

where 𝐧 denotes the inward-pointing unit normal vector. In terms of the fluid velocity potential ϕ, this condition becomes

$$ℨ(y)=\frac{i\rho \omega \phi }{\nabla \phi \cdot 𝐧}=\frac{i\rho \omega \phi }{\frac{\partial \phi }{\partial y}},$$ (43)

assuming variation is normal to the boundary in the *y*-direction. For a single reactive honeycomb layer backed by a rigid wall, the one-dimensional velocity potential satisfies the Helmholtz equation

$$\frac{d^{2}\varphi }{dy^{2}}+k^{2}\varphi (y)=0,\;-b\leq y\leq -h\;\text{and}\;h\leq y\leq b.$$ (44)

The general solution of Eq (44) is given by

$$\varphi (y)=c_{1}\cos(ky)+c_{2}\sin(ky),$$ (45)

leading to the expressions for acoustic pressure *p* and particle velocity *v* as

$$p=i\rho \omega \left[c_{1}\cos(ky)+c_{2}\sin(ky)\right],\;v=k\left[-c_{1}\sin(ky)+c_{2}\cos(ky)\right].$$ (46)

Substituting Eq (46) into the impedance definition (42) yields

$$ℨ(y)=\pm \frac{i\rho \omega }{k\tan[k(b-h)]},\;\text{at}\;y=\pm h.$$ (47)

Using this result in Eq (43), the boundary condition becomes

$$\frac{\partial \phi }{\partial y}-\frac{i\omega \rho }{ℨ(y)}\phi =0,$$ (48)

which can be rewritten in the standard impedance form as

$$\frac{\partial \phi }{\partial y}\pm \xi \phi =0,\;0\leq y\leq L,\;\text{at}\;y=\mp h,$$ (49)

where ξ(k)=ktan[k(b−h)] is the normalized impedance function.

### 3.2 Propagating modes in the inlet and outlet regions

The acoustic modes in the inlet (Region I) and outlet (Region III) of the waveguide, both bounded by rigid surfaces, are formulated using separation of variables. In these regions, the normal component of the particle velocity vanishes on the boundaries due to the rigid-wall conditions:

$$\frac{\partial \phi_{1}}{\partial y}=0,\;y=\pm h,\;-\infty <x<0,$$ (50)

$$\frac{\partial \phi_{3}}{\partial y}=0,\;y=\pm h,\;L<x<\infty .$$ (51)

Solving the Helmholtz equation (4) under the boundary conditions (50)–(51), the velocity potentials in Regions I and III are expressed as modal expansions:

$$\phi_{1}(x,y)=\sum_{n=0}^{\infty }A_{1n}\psi_{n}(y)e^{i\eta_{n}x}+\sum_{n=0}^{\infty }B_{1n}\psi_{n}(y)e^{-i\eta_{n}x},$$ (52)

$$\phi_{3}(x,y)=\sum_{n=0}^{\infty }A_{3n}\psi_{n}(y)e^{i\eta_{n}(x-L)}+\sum_{n=0}^{\infty }B_{3n}\psi_{n}(y)e^{-i\eta_{n}(x-L)},$$ (53)

where $\left\{A_{1n},B_{1n}\right\}$ and $\left\{A_{3n},B_{3n}\right\}$ are modal amplitude coefficients corresponding to the $n^{\text{th}}$ mode. Positive exponential terms correspond to waves traveling in the  + *x* direction, while negative exponentials represent waves traveling in the –*x* direction. The above equations can be compactly written in matrix form as:

$$\phi_{1}=\psi^{t}D_{1}(x)A_{1}+\psi^{t}D_{1}(-x)B_{1},$$ (54)

$$\phi_{3}=\psi^{t}D_{1}(x-L)A_{3}+\psi^{t}D_{1}(L-x)B_{3},$$ (55)

where ψ is the vector of transverse eigenfunctions and the superscript “*t*” indicates the transpose. The diagonal matrix *D*_1_(*x*) is defined as:


$$D_{1}(x)=(\begin{array}{cccc} e^{i\eta_{0}x} & 0 & 0 & \cdots \\ 0 & e^{i\eta_{1}x} & 0 & \cdots \\ 0 & 0 & e^{i\eta_{2}x} & \cdots \\ \vdots & \vdots & \vdots & \ddots \end{array}).$$

The modal amplitude vectors are:


$$A^{t}=[\begin{array}{cccc} A_{0} & A_{1} & A_{2} & \cdots \end{array}],\;B^{t}=[\begin{array}{cccc} B_{0} & B_{1} & B_{2} & \cdots \end{array}].$$

The eigenfunctions of the transverse modes are given by:


$$\psi_{n}(y)=\Lambda_{n}\cos(\tau_{n}y),\;n=0,1,2,\ldots ,$$

where the transverse wavenumbers are $\tau_{n}=\frac{n\pi }{2h}$, and the normalization constants $\Lambda_{n}$ are:


$$\Lambda_{n}=\left\{ \begin{array}{cc} \frac{1}{\sqrt{2h}}, & n=0,\\ \sqrt{\frac{1}{h}}, & n\geq 1. \end{array}\right.$$

These eigen functions satisfy the orthonormality condition:

$$\int_{-h}^{h}\psi_{m}(y)\psi_{n}(y)\;dy=\delta_{mn}.$$ (56)

### 3.3 Propagating modes in the central region and multimodal formulation

The central region contains reactive liners occupying the domains −b≤y<−h and *h*<*y*<*b*, while the interval −h≤y≤h remains unlined. To determine the eigenmodes in this lined central section, the Multi-Model approach is employed. Assuming the field potential in Region II satisfies the liner conditions at y=±h, we introduce the following ansatz:

$$\phi_{2}(x,y)=\sum_{n=0}^{\infty }A_{2n}Z_{n}(y)e^{is_{n}x}.$$ (57)

Substituting Eq (57) into the governing wave Eq (4), we obtain

$$\frac{d^{2}Z_{n}}{dy^{2}}+\gamma_{n}^{2}Z_{n}(y)=0,$$ (58)

where $\gamma_{n}=\sqrt{1-s_{n}^{2}}$ and *Z*_*n*_(*y*) is the unknown transverse function. Imposing the impedance boundary condition from Eq (49), we obtain the following Robin-type boundary conditions:

$$\frac{dZ_{n}}{dy}\pm \xi (k)Z_{n}=0,\;\text{at}\;y=\mp h,$$ (59)

where ξ(k)=ktan[k(b−h)] is the normalized impedance. To construct a solution for *Z*_*n*_(*y*), we define a related eigenvalue problem:

$$\frac{d^{2}}{dy^{2}}\psi_{n}(y)+\alpha_{n}^{2}\psi_{n}(y)=0,$$ (60)

$$\frac{d}{dy}\psi_{n}(y)=0\;\text{at}\;y=\pm h.$$ (61)

Solving Eqs (60)–(61), the eigenfunctions take the orthonormal form:


$$\psi_{n}(y)=\Lambda_{n}\cos\left[\alpha_{n}(y-h)\right],$$

satisfying the orthogonality condition

$$\int_{-h}^{h}\psi_{n}(y)\psi_{m}(y)\;dy=\delta_{mn},$$ (62)

where $\alpha_{n}=\frac{n\pi }{2h}$, for n=0,1,2,… Now express *Z*_*n*_(*y*) as a linear combination of the basis $\psi_{m}(y)$:

$$Z_{n}(y)=\sum_{m=0}^{\infty }A_{mn}\psi_{mn}(y)=\psi_{n}^{T}A_{n},$$ (63)

where $\psi_{n}$ and $A_{n}$ are column vectors of basis functions and modal amplitudes, respectively. Multiplying Eq (58) by $\psi_{n}$ and integrating over y∈[−h,h] gives:

$$\int_{-h}^{h}\psi_{n}\frac{d^{2}Z_{n}}{dy^{2}}\;dy+\gamma_{n}^{2}\int_{-h}^{h}\psi_{n}Z_{n}\;dy=0.$$ (64)

Integrating the first term by parts and applying the boundary conditions (59) and (61), we obtain:

$$\int_{-h}^{h}\psi_{n}\frac{d^{2}Z_{n}}{dy^{2}}\;dy=\xi \psi_{n}(h)Z_{n}(h)+\xi \psi_{n}(-h)Z_{n}(-h)+\int_{-h}^{h}Z_{n}\frac{d^{2}\psi_{n}}{dy^{2}}\;dy.$$ (65)

Substituting Eq (65) into Eq (64) yields:

$$\xi \psi_{n}(h)Z_{n}(h)+\xi \psi_{n}(-h)Z_{n}(-h)+\int_{-h}^{h}Z_{n}\frac{d^{2}\psi_{n}}{dy^{2}}\;dy+\gamma_{n}^{2}\int_{-h}^{h}\psi_{n}Z_{n}\;dy=0.$$ (66)

Since $\psi_{nm}(y)=\Lambda_{m}\cos\left(\frac{m\pi }{2h}y\right)$ is orthogonal, Eq (66) becomes:

$$\xi \psi_{nm}(h)Z_{n}(h)+\xi \psi_{nm}(-h)Z_{n}(-h)-{\left(\frac{m\pi }{2h}\right)}^{2}\int_{-h}^{h}\psi_{nm}(y)Z_{n}(y)\;dy+\gamma_{n}^{2}\int_{-h}^{h}\psi_{nm}(y)Z_{n}(y)\;dy=0.$$ (67)

Using the expansion $Z_{n}(y)=\sum_{p=0}^{\infty }A_{np}\psi_{np}(y)$, Eq (67) becomes:


$$\xi \sum_{p=0}^{\infty }A_{np}\psi_{np}(h)\psi_{nm}(h)+\xi \sum_{p=0}^{\infty }A_{np}\psi_{np}(-h)\psi_{nm}(-h)$$

$$-{\left(\frac{m\pi }{2h}\right)}^{2}\sum_{p=0}^{\infty }A_{np}\delta_{pm}+\gamma_{n}^{2}\sum_{p=0}^{\infty }A_{np}\delta_{pm}=0.$$ (68)

Let the following matrices represent the inner products:


$$\sum_{p=0}^{\infty }A_{np}\psi_{np}(h)\psi_{nm}(h)=N_{1}A,\;\sum_{p=0}^{\infty }A_{np}\psi_{np}(-h)\psi_{nm}(-h)=N_{2}A,$$

where the matrices *N*_1_, *N*_2_, and *N*_3_ are defined as:


$$N_{1}=(\begin{array}{ccc} \psi_{0n}(h)\psi_{0n}(h) & \psi_{0n}(h)\psi_{1n}(h) & \ldots \\ \psi_{1n}(h)\psi_{0n}(h) & \psi_{1n}(h)\psi_{1n}(h) & \ldots \\ \vdots & \vdots & \ddots \end{array}),\;N_{2}=(\begin{array}{ccc} \psi_{0n}(-h)\psi_{0n}(-h) & \psi_{0n}(-h)\psi_{1n}(-h) & \ldots \\ \psi_{1n}(-h)\psi_{0n}(-h) & \psi_{1n}(-h)\psi_{1n}(-h) & \ldots \\ \vdots & \vdots & \ddots \end{array}),$$

$$N_{3}=(\begin{array}{cccc} 0 & 0 & 0 & \cdots \\ 0 & \frac{\pi^{2}}{4h^{2}} & 0 & \cdots \\ 0 & 0 & \frac{4\pi^{2}}{4h^{2}} & \cdots \\ \vdots & \vdots & \vdots & \ddots \end{array}).$$

Thus, Eq (68) can be expressed in matrix form as:

$$\xi N_{1}A+\xi N_{2}A-N_{3}A+\gamma_{n}^{2}IA=0,$$ (69)

Recalling that $\gamma_{n}^{2}=1^{2}-s_{n}^{2}$, Eq (69) can be rewritten as the eigenvalue problem:

$$s_{n}^{2}A=NA,$$ (70)

where


$$N=k^{2}I-N_{3}+\xi N_{1}+\xi N_{2}.$$

Eq (70) shows that $s_{n}^{2}$ are the eigenvalues of the matrix *N*. These eigenvalues and their corresponding eigenvectors can be computed numerically using suitable software. Let *X* be the matrix of eigenvectors of Eq (70). Then, the field potential in the lined region can be written as:

$$\phi_{2}=\psi^{t}XD_{2}(x)A_{2}+\psi^{t}XD_{2}(-x-L)B_{2},$$ (71)

where the diagonal matrix *D*_2_(*x*) is given by:


$$D_{2}(x)=(\begin{array}{cccc} e^{is_{0}x} & 0 & 0 & \cdots \\ 0 & e^{is_{1}x} & 0 & \cdots \\ 0 & 0 & e^{is_{2}x} & \cdots \\ \vdots & \vdots & \vdots & \ddots \end{array}),$$

and the amplitude vectors are


$$A_{2}^{t}=[\begin{array}{ccccc} A_{0} & A_{1} & \cdots & A_{n} & \cdots \end{array}],\;B_{2}^{t}=[\begin{array}{ccccc} B_{0} & B_{1} & \cdots & B_{n} & \cdots \end{array}].$$

Similarly, the acoustic potential in each region of the duct is given by:

$$\phi_{1}=\psi^{t}\left[D_{1}(x)A_{1}+D_{1}(-x)B_{1}\right],$$ (72)

$$\phi_{2}=\psi^{t}\left[XD_{2}(x)A_{2}+XD_{2}(-x-L)B_{2}\right],$$ (73)

$$\phi_{3}=\psi^{t}\left[D_{1}(x-L)A_{3}+D_{1}(L-x)B_{3}\right].$$ (74)

Here, the vectors $\left\{A_{1},A_{2},A_{3}\right\}$ and $\left\{B_{1},B_{2},B_{3}\right\}$ represent the unknown modal amplitudes in each region. For simplicity, we assume $B_{3}=0$, indicating no reflected wave in Region III. The remaining unknowns are determined by applying continuity conditions for pressure and normal velocity at the interfaces *x* = 0 and *x* = *L*. The continuity of acoustic pressure at the interfaces *x* = 0 and *x* = *L* is imposed as follows:

$$\phi_{1}(0,y)=\phi_{2}(0,y),\;-h\leq y\leq h,$$ (75)

$$\phi_{2}(L,y)=\phi_{3}(L,y),\;-h\leq y\leq h.$$ (76)

Similarly, the continuity of normal particle velocities at these interfaces is expressed as:

$$\frac{\partial }{\partial x}\phi_{1}(0,y)=\frac{\partial }{\partial x}\phi_{2}(0,y),\;-h\leq y\leq h,$$ (77)

$$\frac{\partial }{\partial x}\phi_{2}(L,y)=\frac{\partial }{\partial x}\phi_{3}(L,y),\;-h\leq y\leq h.$$ (78)

Substituting Eqs (72)–(74) into the continuity conditions (75)–(78), we obtain:

$$\psi^{t}\left[D_{1}(0)A_{1}+D_{1}(0)B_{1}\right]=\psi^{t}\left[XD_{2}(0)A_{2}+D_{2}(L)B_{2}\right],$$ (79)

$$\psi^{t}\left[XD_{2}(L)A_{2}+XD_{2}(0)B_{2}\right]=\psi^{t}\left[D_{1}(0)A_{3}+D_{1}(0)B_{3}\right],$$ (80)

$$\psi^{t}\left[D_{1}(0)K_{R}A_{1}-D_{1}(0)K_{R}B_{1}\right]=\psi^{t}\left[XK_{Y}D_{2}(0)A_{2}-D_{2}(L)K_{Y}B_{2}\right],$$ (81)

$$\psi^{t}\left[XK_{Y}D_{2}(L)A_{2}-XK_{Y}D_{2}(0)B_{2}\right]=\psi^{t}\left[D_{1}(0)K_{R}A_{3}-D_{1}(0)K_{R}B_{3}\right].$$ (82)

Multiplying Eqs (79)–(82) from the left by ψ and integrating over the interval y∈[−h,h], we derive the following system:

$$A_{1}+B_{1}=X\left(A_{2}+D_{2}(L)B_{2}\right),$$ (83)

$$K_{R}(A_{1}-B_{1})=XK_{Y}\left(A_{2}-D_{2}(L)B_{2}\right),$$ (84)

$$A_{3}+B_{3}=X\left(D_{2}(L)A_{2}+B_{2}\right),$$ (85)

$$K_{R}(A_{3}-B_{3})=XK_{Y}\left(D_{2}(L)A_{2}-B_{2}\right).$$ (86)

Assuming that $B_{3}=0$, we can solve for $B_{2}$ using Eqs (85) and (86):

$$B_{2}=-F^{-1}GD_{2}(L)A_{2},$$ (87)

where


$$F=X+K_{R}^{-1}XK_{Y},\;G=X-K_{R}^{-1}XK_{Y}.$$

Substituting Eq (87) into Eqs (85) and (86), we obtain:

$$2A_{3}=\left(FD_{2}(L)-GF^{-1}GD_{2}(L)\right)A_{2},$$ (88)

$$2A_{1}=\left(F-GD_{2}(L)F^{-1}GD_{2}(L)\right)A_{2},$$ (89)

$$2B_{1}=\left(G-FD_{2}(L)F^{-1}GD_{2}(L)\right)A_{2}.$$ (90)

The transmission and reflection coefficients are then defined as:

$$T(t)=\frac{A_{3}}{A_{1}},$$ (91)

which yields, upon substitution:

$$T(t)=\left(FD_{2}(L)-GF^{-1}GD_{2}(L)\right){\left(F-GD_{2}(L)F^{-1}GD_{2}(L)\right)}^{-1},$$ (92)

and

$$R(r)=\left(G-FD_{2}(L)F^{-1}GD_{2}(L)\right){\left(F-GD_{2}(L)F^{-1}GD_{2}(L)\right)}^{-1}.$$ (93)

Here, *K*_*R*_ and *K*_*Y*_ are diagonal matrices of axial wavenumbers given by:


$$K_{R}=(\begin{array}{ccc} \eta_{0} & 0 & \cdots \\ 0 & \eta_{1} & \cdots \\ \vdots & \vdots & \ddots \end{array}),\;K_{Y}=(\begin{array}{ccc} s_{0} & 0 & \cdots \\ 0 & s_{1} & \cdots \\ \vdots & \vdots & \ddots \end{array}).$$

## 4 Numerical results and discussion

This section presents computational results corresponding to two classes of problems addressed earlier: (i) a semi-infinite duct excited by plane piston sources, and (ii) a configuration involving a fundamental mode incident on a reactive liner cavity to study its scattering characteristics. The numerical findings for both cases are discussed in the following subsections.

### 4.1 Influence of piston excitation on scattering behavior

**Fig 3:**
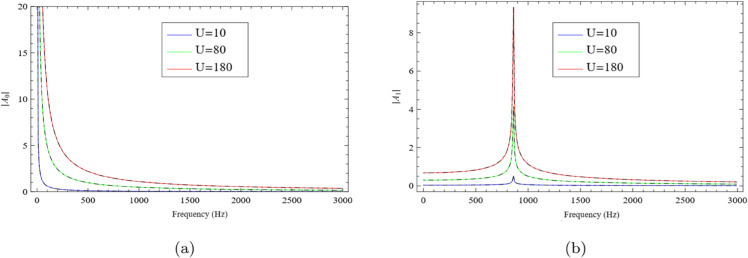
For rigid-rigid configuration: the absolute values of fundamental mode and secondary mode against frequency, where (a) the fundamental mode |*A*_0_| and (b) the secondary mode |*A*_1_|.

The problems excited by plane piston sources are analyzed using both the Mode-Matching Technique and the Multi-Modal Method. The geometric dimensions for the simulations are set as h=0.2m, $h_{1}=0.05\;\text{m}$, and $h_{2}=0.1\;\text{m}$. The speed of sound is taken as c=343.5m/s, and the wavenumber is defined by $k=\frac{2\pi f}{c}$, where the frequency *f* ranges from 1Hz to 3000Hz. The piston velocity *U* is considered at three levels: 10m/s, 80m/s, and 180m/s. The absolute values of the amplitudes for the fundamental mode |*A*_0_| and the first higher-order mode |*A*_1_| are plotted as functions of frequency. Fig 3 demonstrates that the piston velocity has a substantial effect on the amplitudes of the propagating modes. The results from the Mode-Matching approach (solid lines in Fig 3(a) and 3(b)) and the Multi-Modal approach (dashed lines) are in close agreement, indicating strong consistency between the two methods.

In Fig 3(a), at the lowest velocity U=10m/s, the amplitude remains small across the frequency range, indicating weak excitation. When the velocity increases to U=80m/s, the amplitude grows significantly, especially at higher frequencies, reflecting stronger fluid–structure interaction and the onset of resonance behavior. At the highest velocity U=180m/s, a sharp peak emerges within a certain frequency band, suggesting resonance—where the excitation frequency aligns with a natural frequency of the system, resulting in a notable increase in energy transfer. A similar trend is observed in Fig 3(b). For U=10m/s, the amplitude remains low and nearly flat across the spectrum, indicating minimal coupling between the flow and acoustic field. As the velocity rises to U=80m/s, amplitude growth becomes more prominent, especially in the mid-to-high frequency range, signaling stronger energy exchange and potential resonance. At U=180m/s, the response is significantly amplified, with a pronounced peak at higher frequencies—again indicating a resonant condition due to constructive interference between the induced flow excitation and the natural modes of the system.

**Fig 4:**
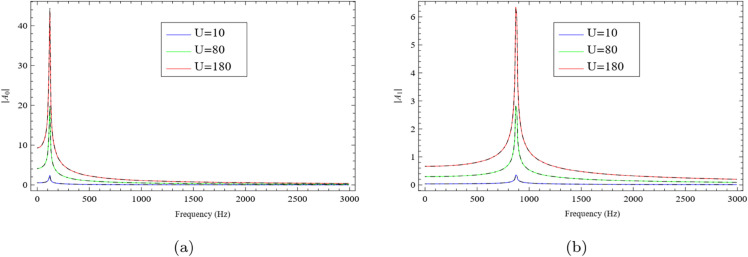
For rigid-impedance configuration: the absolute values of fundamental mode and secondary mode against frequency, where (a) the fundamental mode |*A*_0_| and (b) the secondary mode |*A*_1_|.

Similarly, the results corresponding to the configuration discussed in Sect 2.2 are presented in Fig 4(a) and 4(b). Numerical simulations are conducted using both the Mode-Matching and Multi-Modal methods. The vertical wall heights are taken as h=0.2m, $h_{1}=0.05\;\text{m}$, and $h_{2}=0.1\;\text{m}$. The wavenumber is defined as $k=\frac{2\pi f}{c}$, where the speed of sound is c=343.5m/s, and *f* denotes the frequency in Hertz. The plots illustrate the variation of the fundamental mode amplitude |*A*_0_| and the first higher-order mode amplitude |*A*_1_| as functions of frequency, for three piston velocities: U=10m/s, 80m/s, and 180m/s. As shown in Fig 4(a) and 4(b), both |*A*_0_| and |*A*_1_| increase with piston velocity. The results obtained via the Mode-Matching approach (solid lines) and the Multi-Modal approach (dashed lines) are in excellent agreement, confirming the reliability of the methods.

In Fig 4(a), for the lowest velocity U=10m/s, the amplitude remains small throughout the frequency range, indicating weak excitation and negligible resonance. As the velocity increases to U=80m/s, a gradual rise in amplitude is observed, especially at higher frequencies, suggesting enhanced flow–acoustic interaction. At the highest velocity U=180m/s, a pronounced and narrow peak emerges, indicative of a strong resonant response. This peak signifies a critical frequency band where energy transfer from the flow to the acoustic and structural modes becomes highly efficient. A similar trend is observed in Fig 4(b). At U=10m/s, the amplitude remains low, reflecting weak flow-induced excitation. As the velocity increases to U=80m/s, the amplitude rises, particularly in the mid-to-high frequency range, revealing stronger coupling between the flow and the acoustic field. At U=180m/s, a sharp peak appears over a specific frequency range, clearly indicating a resonant condition. This further emphasizes that higher flow velocities lead to more efficient acoustic excitation and energy exchange within the system.

### 4.2 Impact of lined cavity on scattering behavior

The system is excited by the fundamental duct mode in the inlet region, which undergoes scattering upon encountering the lined cavity. The absolute values of the reflected fundamental mode in the inlet, |*R*_0_|, and the transmitted fundamental mode in the outlet, |*T*_0_|, are plotted as functions of frequency. These two components are of particular interest, as they carry the dominant portion of the incident acoustic energy. For the simulations, the height of the inlet and outlet ducts is taken as h=0.05m, and the length of the lined cavity is set to l=0.1m. The liner depth, given by *b*–*h*, is varied by selecting three values for *b*: 0.1m, 0.2m, and 0.3m. The speed of sound is assumed to be c=343.5m/s, and the wavenumber is defined as $k=\frac{2\pi f}{c}$, where *f* is the frequency in Hertz.

**Fig 5:**
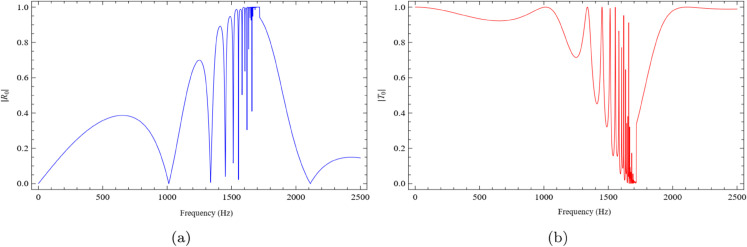
The absolute fundamental reflected and transmitted modes against frequency for b = 0.1m, h = 0.05m and L = 0.1m, where (a) the reflected mode and (b) the transmitted mode.

Fig 5(a) and 5(b) illustrate the frequency-dependent behavior of |*R*_0_| and |*T*_0_|, respectively, for the case b=0.1m. As shown in Fig 5(a), the reflected amplitude begins at a low value and gradually increases with frequency, reaching a maximum within the range 1400Hz≤f≤1700Hz. In contrast, the transmitted amplitude, depicted in Fig 5(b), exhibits an opposite trend, decreasing in the same frequency range. The dips and peaks like variations are primarily attributed to the new propagating modes in the duct and to modal transitions, where the associated eigenvalues shift between imaginary, real, or complex values. Distinct dips in the reflected amplitude are observed at specific frequencies, including f=1020Hz, 1340Hz, 1470Hz, 1550Hz, and 2100Hz. On the other hand, the transmitted amplitude exhibits a marked reduction between f=1600Hz and 1700Hz, indicating strong interaction and possible resonant effects within the lined cavity.

**Fig 6:**
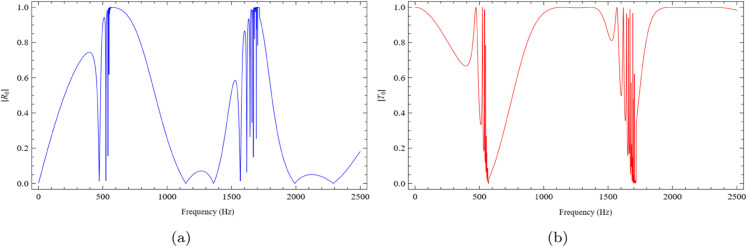
The absolute fundamental reflected and transmitted modes against frequency for b = 0.2m, h = 0.05m and L = 0.1m, where (a) the reflected mode and (b) the transmitted mode.

Similarly, for b=0.2m, the absolute values of the fundamental reflected and transmitted modes are shown in Fig 6. As seen in Fig 6(a), the reflected amplitude exhibits a dip at f=475Hz, followed by an increase at f=515Hz, and then a gradual decrease between f=550Hz and f=1475Hz. The amplitude again increases in the range f=1650Hz to f=1750Hz, and subsequently decreases around f=1800Hz. Conversely, Fig 6(b) presents the transmitted mode, which shows an opposite behavior: a peak at f=475Hz, a drop at f=515Hz, and a rise from f=550Hz to f=1475Hz. A subsequent decrease occurs between f=1650Hz and f=1750Hz, followed by an increase near f=1800Hz. These extrema are consistent with impedance–resonance effects of the liner-backed cavity: the dip in reflection near 475Hz is close to the quarter-wave condition, where the effective surface impedance approaches the duct characteristic impedance, thereby minimizing reflection and producing the corresponding peak in transmission (energy balance R+T≈1). Detuning from this match explains the recovery around 515Hz and the slow variation over 550−1475Hz; features near 1650−1800Hz indicate the next impedance phase crossing and incipient modal energy redistribution. Here *c* is the sound speed and $R=|R_{0}|,T=|T_{0}|$ denote reflection and transmission coefficients, respectively.

**Fig 7:**
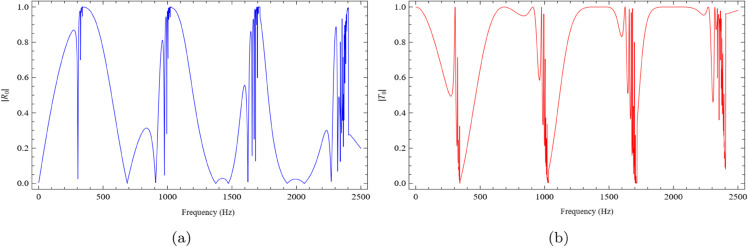
The absolute fundamental reflected and transmitted modes against frequency for b = 0.3m, h = 0.05m and L = 0.1m, where (a) the reflected mode and (b) the transmitted mode.

In the case of b=0.3m, the fundamental reflected and transmitted mode amplitudes are illustrated in Fig 7. Fig 7(a) reveals that the reflected amplitude increases at frequencies f=350Hz, f=1050Hz, and f=2450Hz, while it decreases at f=300Hz, f=700Hz, f=900Hz, within the interval f=1370Hz to f=1470Hz, and again at f=2300Hz. In contrast, Fig 7(b) depicts the transmitted amplitude, which shows an opposite trend, with increases and decreases occurring in inverse correspondence to the reflected mode behavior shown in Fig 7(a). For b=0.3m, the dip around 300Hz aligns with the estimate f≈c/(4b), indicating improved impedance matching and reduced reflection, while the peaks near 350Hz, 1050Hz, and 2450Hz arise near higher-order resonance/cut-on regions where phase and modal conversion reinforce reflection. Conversely, the troughs over 700−900Hz and 1370−1470Hz correspond to anti-resonance windows dominated by the reactive part of the liner impedance, which lowers reflection and shifts energy toward transmission (again consistent with R+T≈1).

**Fig 8:**
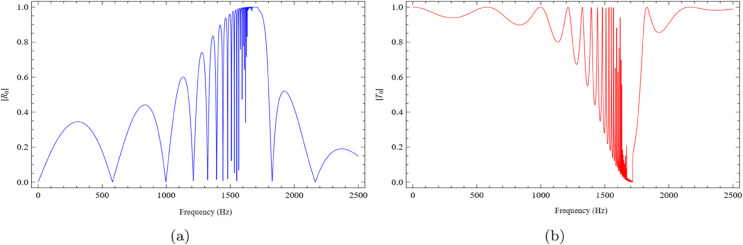
The absolute fundamental reflected and transmitted modes against frequency for b = 0.1m, h = 0.05m and L = 0.2m, where (a) the reflected mode and (b) the transmitted mode.

**Fig 9:**
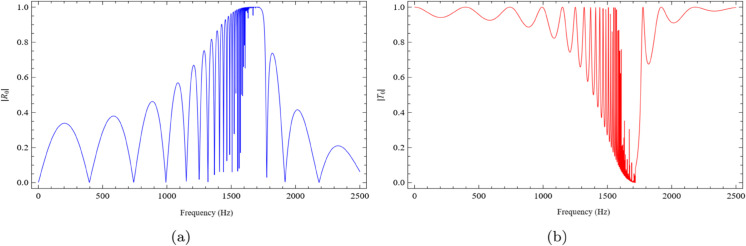
The absolute fundamental reflected and transmitted modes against frequency by increasing cavity length in axial direction for b = 0.1m, h = 0.05m and L = 0.3m, where (a) the reflected mode and (b) the transmitted mode.

Thus, the depth of the cavity plays a significant role in influencing the scattering of acoustic energy, and by adjusting this depth, the performance of the device can be optimized. Figs 8 and 9 present the absolute values of the fundamental reflected and transmitted modes for cavity lengths l=0.2m and l=0.3m, respectively, with fixed values h=0.05m and b=0.1m. The results clearly demonstrate that varying the chamber length leads to noticeable changes in the scattering amplitudes.

## 5 Discussion and conclusion

The study presented in this work investigates the Multi-Modal solution of an acoustic wave scattering problem involving a duct expansion chamber lined with locally reacting materials. The analysis begins with two prototype configurations, both excited by a plane piston source located along the duct wall. The first configuration features entirely rigid boundaries, while the second incorporates impedance linings along the horizontal walls to simulate the effect of locally reacting liners. In both cases, the governing field is described by the Helmholtz equation, subject to either rigid–rigid or rigid–impedance boundary conditions. The associated boundary value problems are formulated and solved using both the Multi-Modal Method and the Mode-Matching Method. Numerical results demonstrate the influence of piston velocity on the scattering characteristics. In particular, it is observed that variations in piston velocity significantly affect both the fundamental and higher-order modal amplitudes. The comparison between the multimodal and Mode matching solutions shows excellent agreement, thereby validating the accuracy and robustness of the proposed formulation. The study is further extended to examine acoustic scattering from a lined expansion chamber comprising two discrete cavity regions. The mathematical modeling of these liner cavities is developed in detail, and a corresponding physical problem is formulated to incorporate their influence on wave propagation. The resulting boundary value problem is solved using the Multi-Modal approach, with simulations performed for various cavity depths and widths. The numerical results reveal that modifications to the cavity geometry–particularly depth and width have a direct impact on the frequency-dependent scattering behavior. Specifically, changes in cavity dimensions lead to a shift in the pass bands and stop bands, thereby altering the effectiveness of the lined expansion chamber across different frequency regimes. These findings suggest that geometric tuning of liner cavities can serve as a powerful design strategy for achieving targeted noise attenuation in specific frequency ranges.

The present study adopts a two dimensional formulation and linear acoustics; consequently, fully three dimensional effects (for example spanwise variations and corner losses) and nonlinear source and liner interactions are not captured. A quiescent medium is assumed, so mean flow or grazing flow and its influence on effective liner impedance and modal dispersion is excluded. The liner is represented via a frequency dependent surface impedance model rather than a resolved poroelastic microstructure. These standard simplifications enable a clear assessment of scattering mechanisms but should be relaxed in future work to quantify flow and liner coupling and fully three dimensional behaviour. Thus, the multimodal method proves to be a highly effective and flexible tool for modeling acoustic wave scattering in ducts with locally reacting liners. It captures complex wave interactions, offers compatibility with impedance boundary modeling, and produces results in close agreement with established mode-matching techniques. This work contributes to the advancement of liner-based noise control systems and provides a framework that can be extended to more complex geometries and real-world acoustic applications.
